# Supplementary material for: TAPBPR mediates peptide dissociation from MHC class I using a leucine lever
Source: eLife. 2018 Nov 28;7:e40126. doi: 10.7554/eLife.40126 (PMC6307860; doi:10.7554/eLife.40126)
Supplement: Figure 5—source data 1. — Soluble TAPBPRWT or soluble TAPBPRØloop were incubated with the indicated LABScreen single antigen HLA class I beads (from One Lambda, Canoga Park, California) for 60 min at RT. After washing 3 times in PBS, bound TAPBPR was detected using the TAPBPR-specific mAb PeTe4 and a goat anti-mouse PE-conjugated secondary antibody. Samples were analysed using the Luminex Fluoroanalyser system. The table shows the MFI of PeTe4 staining ± SD for the indicated HLA beads incubated in the absence and presence of 1 µM of TAPBPRWT or TAPBPRØloop. The expression of MHC I (detected using W6/32) provided by One Lambda for the specific lot of LABScreen beads used is included as a control. [file elife-40126-fig5-data1.docx]

**Figure 5 – Source data 1: Binding of TAPBPR to the individual HLA molecules found in HeLaM cells**

|  | no TAPBPR | TAPBPRWT | TAPBPRØloop | W6/32 |
| --- | --- | --- | --- | --- |
| A*68:02 | 418.3 (± 31.1) | 14461 ±(139) | 2576.3 ±(19.6) | 23344 |
| B*15:03 | 6 (± 10.4) | 21.7 ±(2.3) | 17.3 ±(1.5) | 23670 |
| C*12:03 | 146 (±16.8) | 249 ±(12) | 175 ±(14.8) | 23814 |

Soluble TAPBPRWT or soluble TAPBPRØloop were incubated with the indicated LABScreen single antigen HLA class I beads (from One Lambda, Canoga Park, California) for 60 min at RT. After washing 3 times in PBS, bound TAPBPR was detected using the TAPBPR specific mAb PeTe4 and a goat anti-mouse PE-conjugated secondary antibody. Samples were analysed using the Luminex Fluoroanalyser system. The table shows the MFI of PeTe4 staining +/- SD for the indicated HLA beads incubated in the absence and presence of 1 µM of TAPBPRWT or TAPBPRØloop. The expression of MHC I (detected using W6/32) provided by One Lambda for the specific lot of LABScreen beads used is included as a control.
