# Supplementary material for: TAPBPR mediates peptide dissociation from MHC class I using a leucine lever
Source: eLife. 2018 Nov 28;7:e40126. doi: 10.7554/eLife.40126 (PMC6307860; doi:10.7554/eLife.40126)
Supplement: Transparent reporting form [file elife-40126-transrepform.docx]

***eLife’s* transparent reporting form**

We encourage authors to provide detailed information *within their submission* to facilitate the interpretation and replication of experiments. Authors can upload supporting documentation to indicate the use of appropriate reporting guidelines for health-related research (see [EQUATOR Network](http://www.equator-network.org/%20)), life science research (see the [BioSharing Information Resource](https://biosharing.org/)), or the [ARRIVE guidelines](http://www.plosbiology.org/article/info:doi/10.1371/journal.pbio.1000412) for reporting work involving animal research. Where applicable, authors should refer to any relevant reporting standards documents in this form.

If you have any questions, please consult our Journal Policies and/or contact us: [editorial@elifesciences.org](mailto:editorial@elifesciences.org).

**Sample-size estimation**

- You should state whether an appropriate sample size was computed when the study was being designed
- You should state the statistical method of sample size computation and any required assumptions
- If no explicit power analysis was used, you should describe how you decided what sample (replicate) size (number) to use

Please outline where this information can be found within the submission (e.g., sections or figure legends), or explain why this information doesn’t apply to your submission:

A Power calculation was not performed before the study design. However our assays are based on flow cytometry therefore data was collected on 10,000 cells and include minimum of three technical repeats. Furthermore, standard sample sizes (5x10^8^ cells) were used for immunopeptidomic work.

**Replicates**

- You should report how often each experiment was performed
- You should include a definition of biological versus technical replication
- The data obtained should be provided and sufficient information should be provided to indicate the number of independent biological and/or technical replicates
- If you encountered any outliers, you should describe how these were handled
- Criteria for exclusion/inclusion of data should be clearly stated
- High-throughput sequence data should be uploaded before submission, with a private link for reviewers provided (these are available from both GEO and ArrayExpress)

Please outline where this information can be found within the submission (e.g., sections or figure legends), or explain why this information doesn’t apply to your submission:

With the exception of the data shown in Figure 5, all experiments were performed at least 3 times and this information can be found in the figure legends. For our assays which are based on flow cytometry (Figure 3, 4, 5, 6) data was collected on 10,000 cells and at least three independent technical repeats were performed for each experiments. Any outliners are included in the figures.

The immunopeptidome data shown in figure 5 includes analysis on 2 biological replicate of 5x10^8^ cells per sample. Isolated HLA peptides were analysed in five technical replicates. Furthermore, the data from a third biological repeat is included in the supplementary figures. Figure 5 – figure supplement 1 shows the technical reproducibility of the data in Figure 5. Information regarding replicates and exclusion criteria is provided in the methods.

**Statistical reporting**

- Statistical analysis methods should be described and justified
- Raw data should be presented in figures whenever informative to do so (typically when N per group is less than 10)
- For each experiment, you should identify the statistical tests used, exact values of N, definitions of center, methods of multiple test correction, and dispersion and precision measures (e.g., mean, median, SD, SEM, confidence intervals; and, for the major substantive results, a measure of effect size (e.g., Pearson's r, Cohen's d)
- Report exact p-values wherever possible alongside the summary statistics and 95% confidence intervals. These should be reported for all key questions and not only when the p-value is less than 0.05.

Please outline where this information can be found within the submission (e.g., sections or figure legends), or explain why this information doesn’t apply to your submission:

The statistical analysis included for our flow cytometry based assays (Figure 2,3,4,6,7) is described in the figure legends (mean fluorescent intensity +/-SD, p value based on unpaired two-tailed t-tests). A typical example of the raw data is shown in the histograms for these assays in the main figures and a graph summarizing the results from the technical replicates is also included in the main figures.

Information regarding the statistical methods used for the immunopeptdomics data can be found in the methods. Raw data (full peptide lists) are provided in the auxillary files. Exact P-values are provided in the auxillary files.

(For large datasets, or papers with a very large number of statistical tests, you may upload a single table file with tests, Ns, etc., with reference to sections in the manuscript.)

**Group allocation**

- Indicate how samples were allocated into experimental groups (in the case of clinical studies, please specify allocation to treatment method); if randomization was used, please also state if restricted randomization was applied
- Indicate if masking was used during group allocation, data collection and/or data analysis

Please outline where this information can be found within the submission (e.g., sections or figure legends), or explain why this information doesn’t apply to your submission:

We do not have any data in our manuscript which involved group allocation.

**Additional data files (“source data”)**

- We encourage you to upload relevant additional data files, such as numerical data that are represented as a graph in a figure, or as a summary table
- Where provided, these should be in the most useful format, and they can be uploaded as “Source data” files linked to a main figure or table
- Include model definition files including the full list of parameters used
- Include code used for data analysis (e.g., R, MatLab)
- Avoid stating that data files are “available upon request”

Please indicate the figures or tables for which source data files have been provided:

We have provided full peptide lists of the immunopeptidomic analysis used to generate figure 5. This is provided as 11 excel files (auxillary files). This data has also been uploaded to dryad. <https://datadryad.org/review?doi=doi:10.5061/dryad.p5k0156>
